# Editorial: Physical activity behavior, obesity, and stress as crucial sources of health issues in stressful occupations

**DOI:** 10.3389/fendo.2023.1323360

**Published:** 2023-11-10

**Authors:** Katie M. Heinrich, Filip Kukić, Brittany S. Hollerbach

**Affiliations:** ^1^ Center for Fire, Rescue & EMS Health Research, NDRI-USA, Inc., New York, NY, United States; ^2^ Department of Kinesiology, Kansas State University, Manhattan, KS, United States; ^3^ Faculty of Physical Education and Sports, University of Banja Luka, Banja Luka, Bosnia and Herzegovina

**Keywords:** police officers, firefighters, healthcare workers, nurses, muscle, fitness

Occupations at the front line of public safety, security, and health (e.g., military, police, firefighters, medical professionals, social workers, etc.) have highly stressful mental and physical demands (1), all of which can negatively affect the endocrine system (2). These stressors also negatively affect worker health and performance, which impacts job effectiveness and quality of life (3). Compounding the effects of stress, low levels of physical activity (i.e., below the 150-minute minimum of moderate aerobic activity recommended per week) (4, 5), poor physical fitness (6), and having obesity further exacerbate negative health effects. Insufficient muscle strengthening activity (i.e., <2 days of full-body muscle strengthening activities per week) (5) can result in low muscle mass that lessens movement potential and increases sarcopenia risk (7). Conversely, completing sufficient aerobic and muscle strengthening physical activities each week and maintaining a healthy body composition help mitigate the negative effects of stress (8) and can improve endocrine responses (9).

This Research Topic was designed to focus on mechanisms and endocrine system effects of physical activity behaviors, obesity and stress on the health and performance of workers in stressful occupations. Four papers focus on the stressful tactical professions of firefighting and law enforcement. Ras et al. examine how different tests of physical fitness are related to musculoskeletal injuries and discomfort among full-time firefighters in Cape Town, South Africa. In another study (Ras et al.), they report on relationships between key aspects of physical fitness, cardiovascular health, musculoskeletal health and occupational performance. Among police officers in the Midwestern United States (US), better physical readiness was predicted by higher estimated maximal oxygen consumption (i.e., VO2max) and lower bodyfat percentage in a study by Dicks et al. With a focus on prevention, Hershey et al. share their aims for addressing low levels of physical fitness and high levels of obesity among firefighter recruits via a protocol paper for the usability testing and piloting of a healthy lifestyle app, *Surviving and Thriving*, in the US. App content addresses nutrition, physical activity, sleep, and resilience. Overall, data from these tactical populations shows the value and necessity of maintaining high levels of comprehensive physical fitness and optimal body composition for occupational performance and cardiovascular and musculoskeletal health, as well as for injury prevention.

Two other papers focus on stressful public health and medical populations. In their study of healthcare workers in the Gaza Strip, Younis et al. examine both the high prevalence rates for overweight and obesity along with key associated factors, including chronic disease conditions. Zhao et al. of nurses in South China, examines differences by day versus night shifts in indicators for abnormal liver and kidney function and dyslipidemia. Finally, a dietary supplementation and exercise intervention study among males with obesity by Saeidi et al. finds promise for combining high intensity functional training (HIFT) and spinach-derived thylakoid supplementation on selected adipokines and insulin resistance.

Overall, this body of research adds to the diversity of obesity-related research that is required to address the dynamic relationships between physical activity, obesity and stress, including the underlying mechanisms and endocrine system effects.

## Author contributions

KH: Writing – original draft, Writing – review & editing. FK: Writing – original draft, Writing – review & editing. BH: Writing – review & editing.
